# Morphological characteristics of lumbar vertebral bodies and regional distribution patterns of bone mineral density: a CT study

**DOI:** 10.3389/fphys.2026.1820186

**Published:** 2026-05-14

**Authors:** Xiaoteng Li, Fengzi Lv, Xin Tang, Peng Jia, Yang Gao

**Affiliations:** 1 Department of Orthopedic Trauma, The First Affiliated Hospital of Dalian Medical University, Dalian, Liaoning, China; 2 Department of Spinal Orthopedics II, Zhengzhou Orthopedic Hospital, Zhengzhou, Henan, China; 3 Department of Anesthesiology, Henan Provincial People’s Hospital, Zhengzhou, Henan, China; 4 Department of Orthopedics, The Second Hospital of Shandong Province, Jinan, Shandong, China

**Keywords:** bone mineral density, CT value, lumbar vertebrae, morphology, osteoporotic fracture

## Abstract

**Objective:**

To establish a comprehensive database of the macroscopic morphology and spatial distribution of bone mineral density (BMD) in lumbar vertebral bodies, analyze morphological variation patterns of lumbar structures, and provide reference data for spinal biomechanical modeling and clinical surgery.

**Methods:**

A total of 100 healthy volunteers (50 males, 50 females; age range, 20–70 years) who underwent lumbar spine CT evaluation at the Department of Health Examination, Zhengzhou Orthopedic Hospital between September 2023 and September 2025 were retrospectively enrolled in this study. On CT images, seven morphological parameters (anterior, middle, and posterior vertebral body heights; superior and inferior endplate widths; anterior one-third and posterior one-third cortical thickness of the superior endplate) and CT values in 15 different regions (the vertebral body sagittal plane divided into upper, middle, and lower thirds; the horizontal plane divided into anterior, posterior, left, and right quadrants) were measured for each vertebral body from L1 to L5.

**Results:**

① Morphology: Superior and inferior endplate widths increased progressively from L1 to L5. The relationship between anterior and posterior vertebral body heights showed posterior height > anterior height at L1 and L2, showed no statistically significant difference at L3, and anterior height > posterior height at L4 and L5. The cortical thickness of the anterior one-third of the superior endplate was significantly smaller according to the posterior one-third (P < 0.05). ② CT values: Within vertebral bodies, CT attenuation values showed a gradual increase from the upper one-third to the lower one-third regions. Comparison across segments roughly followed the pattern L1 > L2 > L5 > L3 > L4. Across all segments, the CT value in the anterosuperior region of the vertebral body (anterosuperior region) was the lowest.

**Conclusion:**

The morphology of lumbar vertebral bodies demonstrates a consistent morphological trend from L1 to L5 which may be associated with increasing axial loads and contribute to physiological lordosis. There is significant heterogeneity in the distribution of BMD within the vertebral body, with the anterosuperior region identified as a “stress-weak zone, ” highly consistent with the predilection site for clinical compression fractures. The morphological and densitometric database established in this study can provide reference data for spinal surgery planning, implant design, and biomechanical research.

## 1 Introduction

The lumbar spine is a major load-bearing component of the axial skeleton, and its physiological and pathological states strongly influence spinal stability and function (Tian et al., 2025). Lumbar degenerative diseases, osteoporosis, trauma, tumors, and deformities are primary causes of low back pain, neurological dysfunction, and even disability in adults, imposing an estimated annual direct economic burden of over $100 billion worldwide-a figure that continues to rise with population aging-in addition to substantial personal burdens (Wei et al., 2026). Contemporary spinal surgery has entered the era of precision medicine; the successful execution of procedures, whether internal fixation, osteotomy, interbody fusion, minimally invasive procedures, or vertebral augmentation, relies on accurate and individualized assessment of vertebral body morphology (e.g., endplate dimensions, vertebral body heights, and cortical thickness) and regional bone mineral density distribution within the vertebra.

Morphological parameters of the vertebral body form the basis for determining its biomechanical properties. For example, endplate width is crucial for selecting appropriate interbody cage sizes (Chen et al., 2024). Pedicle screw placement, including screw diameter, entry point, depth, and trajectory, is primarily guided by pedicle morphology; however, the optimal trajectory is also influenced by intravertebral bone mineral density (BMD) distribution, as regional variations in bone quality (e.g., lower density in the anterosuperior region) affect screw purchase and pullout strength. Vertebral body height, together with intervertebral disc height, contributes to spinal curvature (Nişancı and Yıldız, 2026); a recent MRI-based study confirmed that disc height decreases significantly in degenerative conditions while vertebral body height remains preserved, underscoring their distinct yet synergistic roles in sagittal alignment (Nişancı and Yıldız, 2026). Although existing literature describes lumbar vertebral morphology, most studies focus on posterior structures like the pedicle (Arockiaraj et al., 2025; Ma et al., 2025; Moncada-Habib et al., 2025), with insufficient systematic and detailed research on the vertebral body itself, particularly the cortical bone thickness of the endplate. Utilizing clinical CT data for bone quality assessment has become a research hotspot. Measuring Hounsfield units (HU) of the lumbar vertebral cancellous bone allows for efficient, non-invasive acquisition of regional BMD information (Liu et al., 2022; Zhang et al., 2023). Numerous studies confirm a good correlation between vertebral CT values and dual-energy X-ray absorptiometry (DXA) T-scores, effectively predicting complications such as screw loosening, cut-out, and vertebral fractures after internal fixation (Elarjani et al., 2021). However, current CT value measurements are often confined to one or a few regions of interest in the mid-vertebral cancellous bone, potentially overlooking the inherent spatial heterogeneity of BMD within the vertebral body.

Vertebral morphology and bone quality are not independent but rather an interrelated binary system collectively determining mechanical performance. A morphologically wide vertebral body with extremely low BMD cannot provide a reliable foundation for fixation; conversely, a vertebra with acceptable BMD but a weak endplate cortex may fail prematurely under stress. Currently, studies combining detailed morphological measurements with multi-dimensional BMD distribution analysis are scarce. Elarjani et al. (2021) confirmed a positive correlation between mid-sagittal CT values and DXA T-scores, useful for rapid preoperative bone quality assessment, but did not address regional intravertebral differences. Ma et al. (2024) utilized 3D U-Net neural network technology to obtain vertebral morphological parameters from low-dose chest CT, revealing sex- and age-related trends in vertebral height ratios in a Chinese population, highlighting the importance of establishing ethnicity- and site-specific reference data.

In summary, this study aims to establish a comprehensive database encompassing lumbar macroscopic morphology, micro-morphology, and spatial BMD distribution. By elucidating the distribution patterns of lumbar vertebral bodies from the perspectives of individual vertebral morphology and intravertebral BMD distribution, we seek to provide more accurate input parameters for spinal biomechanical modeling and offer localized reference data for clinical surgery.

## 2 Methods

### 2.1 Study population

A total of 100 healthy volunteers who underwent lumbar spine CT examination at the Department of Health Examination, Zhengzhou Orthopedic Hospital between September 2023 and September 2025 were retrospectively collected. This study was approved by the Ethics Committee of Zhengzhou Orthopedic Hospital (Approval No. 2025KY10001), and all volunteers provided written informed consent. This study was conducted in accordance with the Declaration of Helsinki.

Inclusion criteria were: ① age 20–70 years; ② undergoing standard lumbar spine CT examination; ③ no history of lumbar surgery or trauma. Exclusion criteria were: ① presence of vertebral fracture on CT images (assessed using semiquantitative method (Elarjani et al., 2021)); ② congenital vertebral deformity or acquired short vertebral body; ③ spinal metal implants; ④ use of medications affecting bone metabolism (e.g., corticosteroids) or presence of diseases affecting bone metabolism (e.g., multiple myeloma, rheumatoid arthritis, ankylosing spondylitis, systemic lupus erythematosus, metabolic or endocrine disorders, bone tumors); ⑤ adult degenerative scoliosis or severe osteophytosis.

### 2.2 CT scanning protocol

A German Siemens Somatom Definition 64 AS+ spiral CT scanner was used. Scanning parameters were: slice thickness 3.0 mm, detector collimation 128 mm×0.6 mm, pitch 0.8, scan angle 0°, tube voltage 120 kV, with spiral volumetric scanning. To ensure consistency of CT value measurements across subjects, all scans were performed using the same scanner with identical tube voltage (120 kV) and without contrast enhancement, as contrast agents can significantly alter Hounsfield unit measurements and reduce the accuracy of bone density assessment (Abdullayev et al., 2018).

### 2.3 Image reconstruction

Raw data underwent thin-slice reconstruction: slice thickness 1 mm, slice interval 1 mm, reconstruction algorithm B60s sharp, bone window. Reconstructed images were transferred to a syngo multimodality workplace VE36A workstation for multiplanar reformation (MPR). All image reconstructions were performed using a consistent reconstruction kernel (B60s sharp) and slice thickness (1 mm), as variation in reconstruction parameters has been shown to influence CT value measurements (Genant et al., 1993).

### 2.4 Parameter measurements

#### 2.4.1 Vertebral morphological parameters

**Figure 1:**
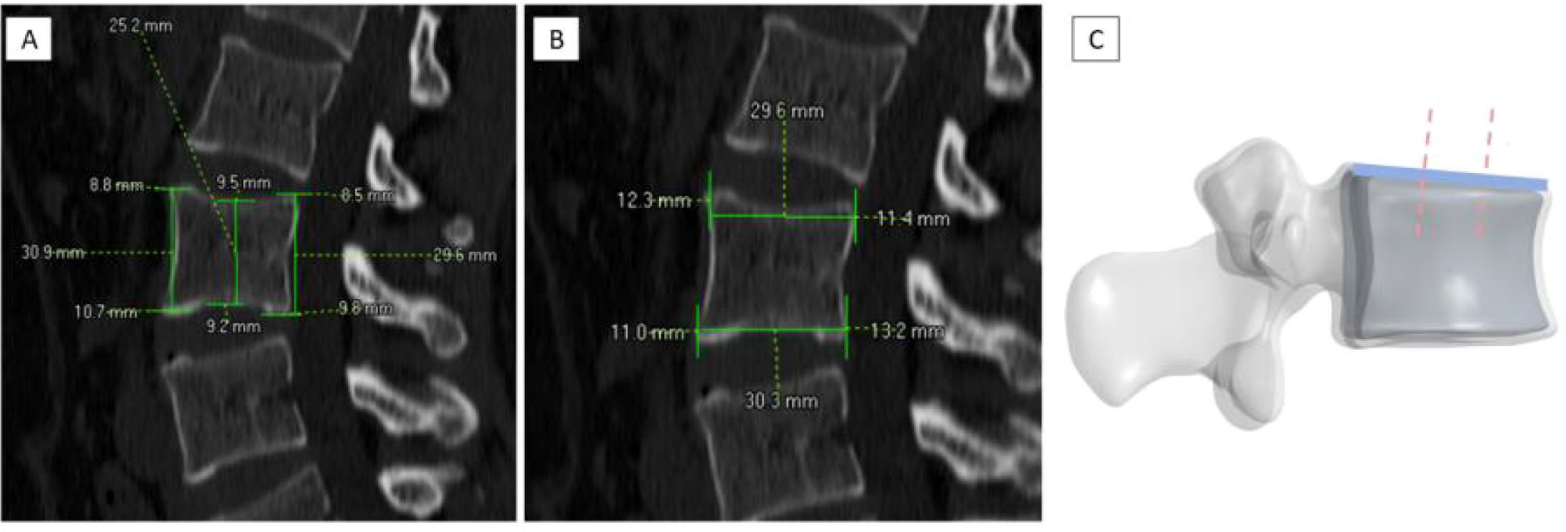
Schematic diagram of morphological measurement indicators of the lumbar vertebral body. **(A)** Anterior vertebral body height (VBHa), middle vertebral body height (VBHm), and posterior vertebral body height (VBHp). **(B)** Superior endplate width (EPWu) and inferior endplate width (EPWl). **(C)** Anterior one-third cortical thickness of the superior endplate (EPTA) and posterior one-third cortical thickness of the superior endplate (EPTP).

The following parameters were measured on mid-sagittal images (**Figure 1**):

① Anterior vertebral body height (VBHa): distance from the anterior edge of the superior endplate to the anterior edge of the inferior endplate.
② Middle vertebral body height (VBHm): distance from the midpoint of the superior endplate to the midpoint of the inferior endplate.
③ Posterior vertebral body height (VBHp): distance from the posterior edge of the superior endplate to the posterior edge of the inferior endplate.
④ Superior endplate width (EPWu): horizontal distance between the outermost lateral edges of the superior endplate.
⑤ Inferior endplate width (EPWl): horizontal distance between the outermost lateral edges of the inferior endplate.
⑥ Anterior 1/3 cortical thickness of superior endplate (EPTA): The superior endplate was divided into three equal parts, and the vertical cortical bone thickness was measured in the anterior third.
⑦ Posterior 1/3 cortical thickness of superior endplate (EPTP): The thickness was measured in the posterior third.

All morphological parameters were measured on mid-sagittal and mid-coronal planes following previously established protocols (Liu et al., 2018; Han et al., 2023). To minimize measurement error, all measurements were performed by a single trained observer (Li Xiaoteng) who was blinded to subject characteristics. Intra-observer reproducibility was assessed by repeated measurements of 20 randomly selected vertebrae after a 2-week interval, yielding intraclass correlation coefficients (ICC) > 0.90 for all parameters.

Each parameter was measured three times, and the average value was calculated. A schematic diagram of the seven vertebral morphological measurement indicators is shown in **Figure 1**.

#### 2.4.2 Measurement of CT values in different vertebral regions

**Figure 2:**
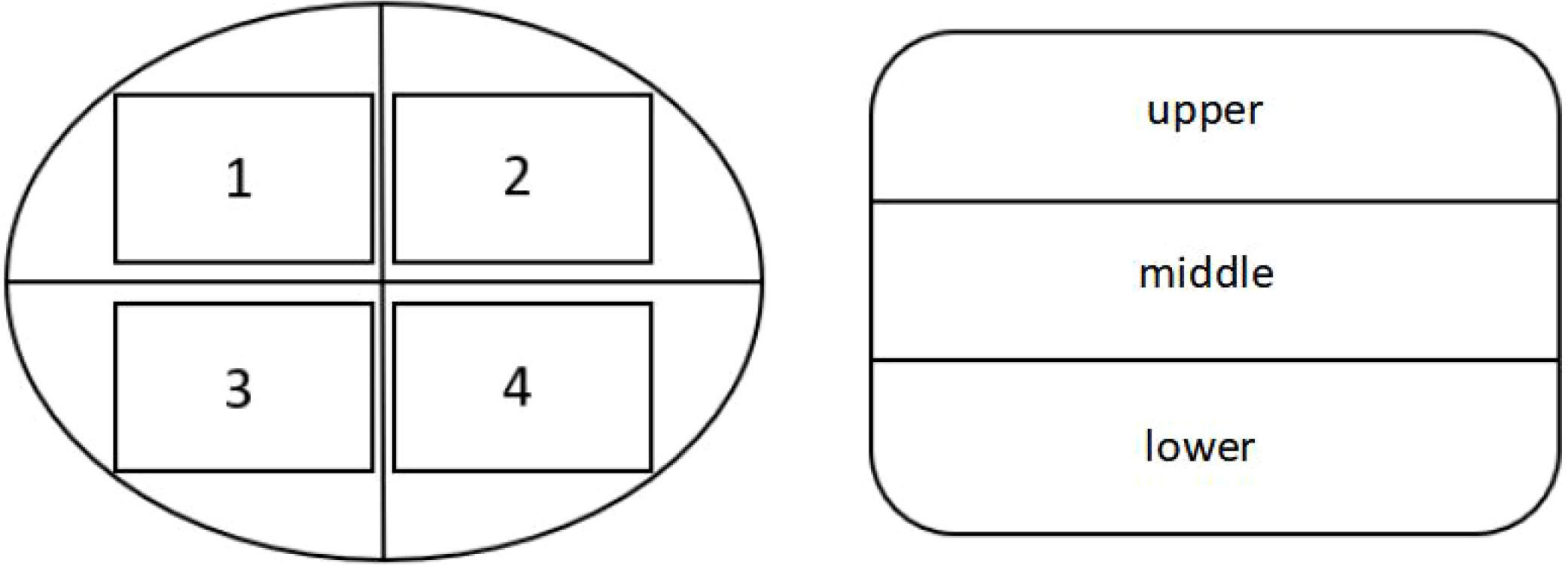
Left image shows the four quadrants of the vertebral body in the horizontal plane; right image shows the three parts of the vertebral body in the sagittal plane.

**Figure 3:**
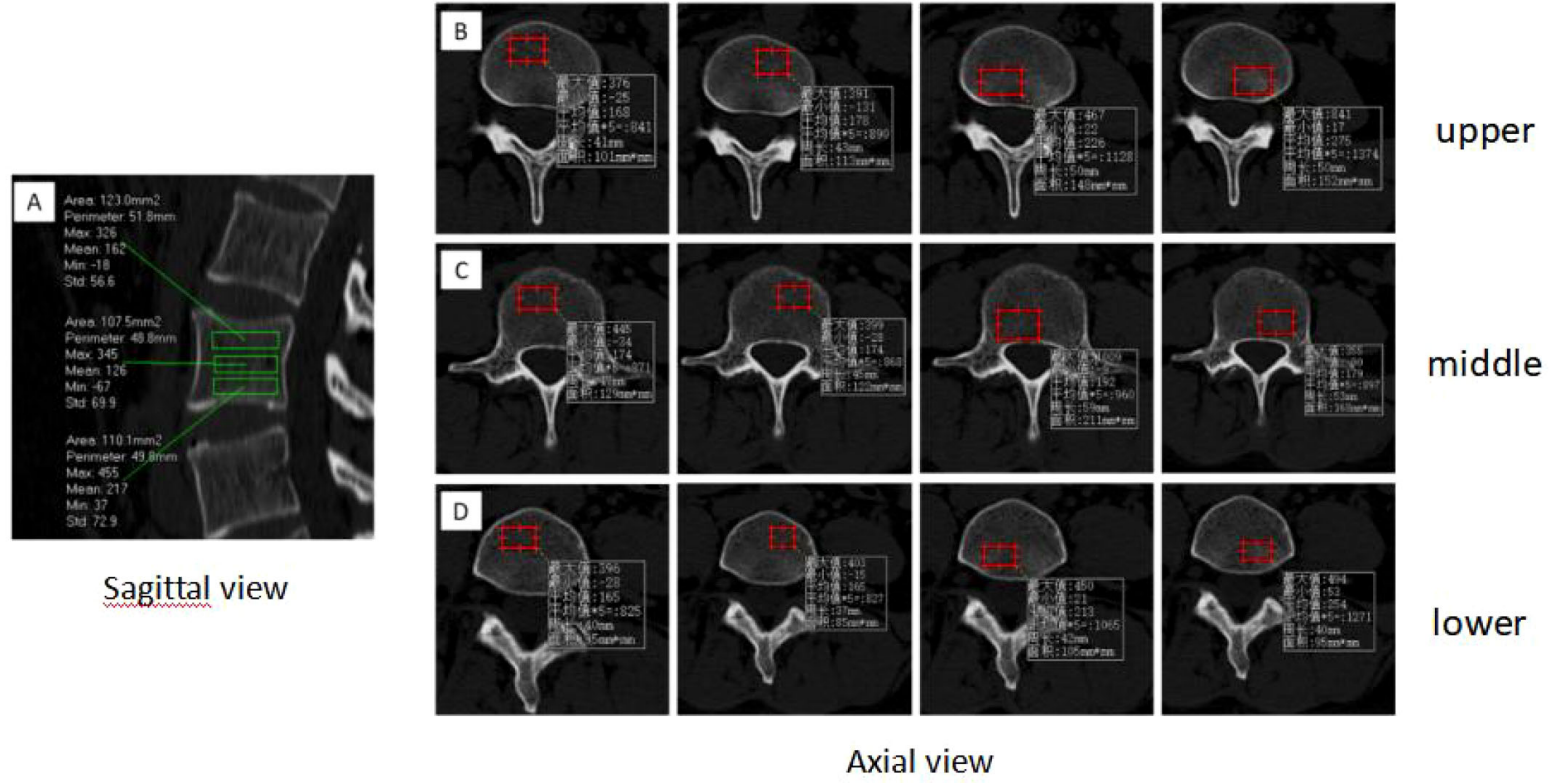
Schematic diagram of CT HU value measurement in different regions within the lumbar vertebral body. **(A)** The lumbar vertebral body is equally divided into upper, middle, and lower thirds in the sagittal plane, and CT values are measured in the three regions. **(B)** At the upper-third level, the horizontal plane is divided into four quadrants, and CT values are measured in the four quadrants. **(C)** At the middle-third level, the horizontal plane is divided into four quadrants, and CT values are measured in the four quadrants. **(D)** At the lower-third level, the horizontal plane is divided into four quadrants, and CT values are measured in the four quadrants.

According to the method of Banse et al. (2001), each vertebral body was divided into upper, middle, and lower thirds in the sagittal plane and into four quadrants (left anterior, right anterior, left posterior, right posterior) in the horizontal plane, yielding 15 regions of interest (ROIs) (**Figures 2**, **3**). This standardized partitioning protocol has been validated in multiple previous studies of intravertebral BMD distribution (Banse et al., 2001; Li et al., 2011; Wang et al., 2022). A circular or elliptical ROI (area approximately 1 cm²) was placed in each region, carefully avoiding the bone cortex, basivertebral foramen, and any visible osteophytes or calcifications. The CT value (Hounsfield unit, HU) was recorded as the mean value within the ROI. To ensure measurement consistency, ROI size and shape were kept as uniform as possible across all regions and all vertebral levels (Wang et al., 2022). Each region was measured three times, and the average value was calculated.

### 2.5 Statistical analysis

Statistical analysis was performed using SPSS software version 23.0. Measurement data are presented as mean ± standard deviation. The normality of the data distribution was assessed using the Shapiro–Wilk test. Homogeneity of variances was evaluated using Levene’s test. For variables that followed a normal distribution and had homogeneous variances, comparisons between two groups were conducted using the independent samples t-test, and comparisons among multiple groups were performed using one-way analysis of variance (ANOVA), with *post-hoc* multiple comparisons adjusted by the Bonferroni correction. If the data deviated significantly from normality or homogeneity of variances, the non-parametric Mann–Whitney U test (for two groups) or Kruskal–Wallis H test (for multiple groups) was used instead. A P-value < 0.05 was considered statistically significant.

## 3 Results

### 3.1 General characteristics

A total of 100 volunteers were enrolled, comprising 50 males and 50 females, aged 20 to 70 years. The distribution across age groups is shown in **Table 1**.

**Table 1:** Demographic data of study participants (mean ± SD).

Age group (years)	Mean age (years)	Male (n)	Female (n)
20-30	25.1 ± 3.4	10	10
31-40	35.6 ± 2.9	8	12
41-50	45.2 ± 2.5	6	14
51-60	55.7 ± 3.1	13	7
61-70	65.4 ± 2.3	9	11

### 3.2 Results of lumbar vertebral body morphological measurements

The measurement results for the seven morphological parameters of L1 to L5 vertebral bodies are presented in **Table 2**. The widths of both the superior and inferior endplates showed a progressive increase from L1 to L5. Analysis of vertebral body heights revealed: posterior height > anterior height at L1 and L2; approximately equal anterior and posterior heights at L3; and anterior height > posterior height at L4 and L5. The cortical thickness of the anterior 1/3 of the superior endplate (0.8 ± 0.1 mm) was consistently smaller than that of the posterior 1/3 (0.9 ± 0.2 mm) at all levels, a difference that was statistically significant (P < 0.05).

**Table 2:** Morphological parameter measurements (mean ± SD, mm).

Level	VBHa	VBHm	VBHp	EPWu	EPWl	EPTA	EPTP
L1	25.1 ± 1.4	23.5 ± 1.3	27.2 ± 1.3	41.8 ± 1.0	44.8 ± 1.1	0.8 ± 0.1	0.9 ± 0.2
L2	26.5 ± 1.3	24.3 ± 1.4	28.1 ± 2.6	43.4 ± 0.7	45.7 ± 0.8	0.8 ± 0.1	1.0 ± 0.2
L3	27.1 ± 1.4	24.4 ± 1.2	27.3 ± 2.2	45.2 ± 0.6	48.7 ± 0.8	0.8 ± 0.2	1.0 ± 0.2
L4	27.6 ± 0.7	23.7 ± 1.2	26.3 ± 2.1	47.7 ± 0.8	51.3 ± 1.7	0.8 ± 0.2	0.9 ± 0.2
L5	27.7 ± 0.6	23.0 ± 0.6	24.6 ± 2.5	50.7 ± 2.4	52.4 ± 0.6	0.8 ± 0.2	1.0 ± 0.2

VBHa, Anterior vertebral body height; VBHm, Middle vertebral body height; VBHp, Posterior vertebral body height; EPWu, Superior endplate width; EPWl, Inferior endplate width; EPTA, Anterior 1/3 cortical thickness of superior endplate; EPTP, Posterior 1/3 cortical thickness of superior endplate.

### 3.3 Results of CT value measurements in different intravertebral regions

#### 3.3.1 Comparison of sagittal partitions

**Figure 4:**
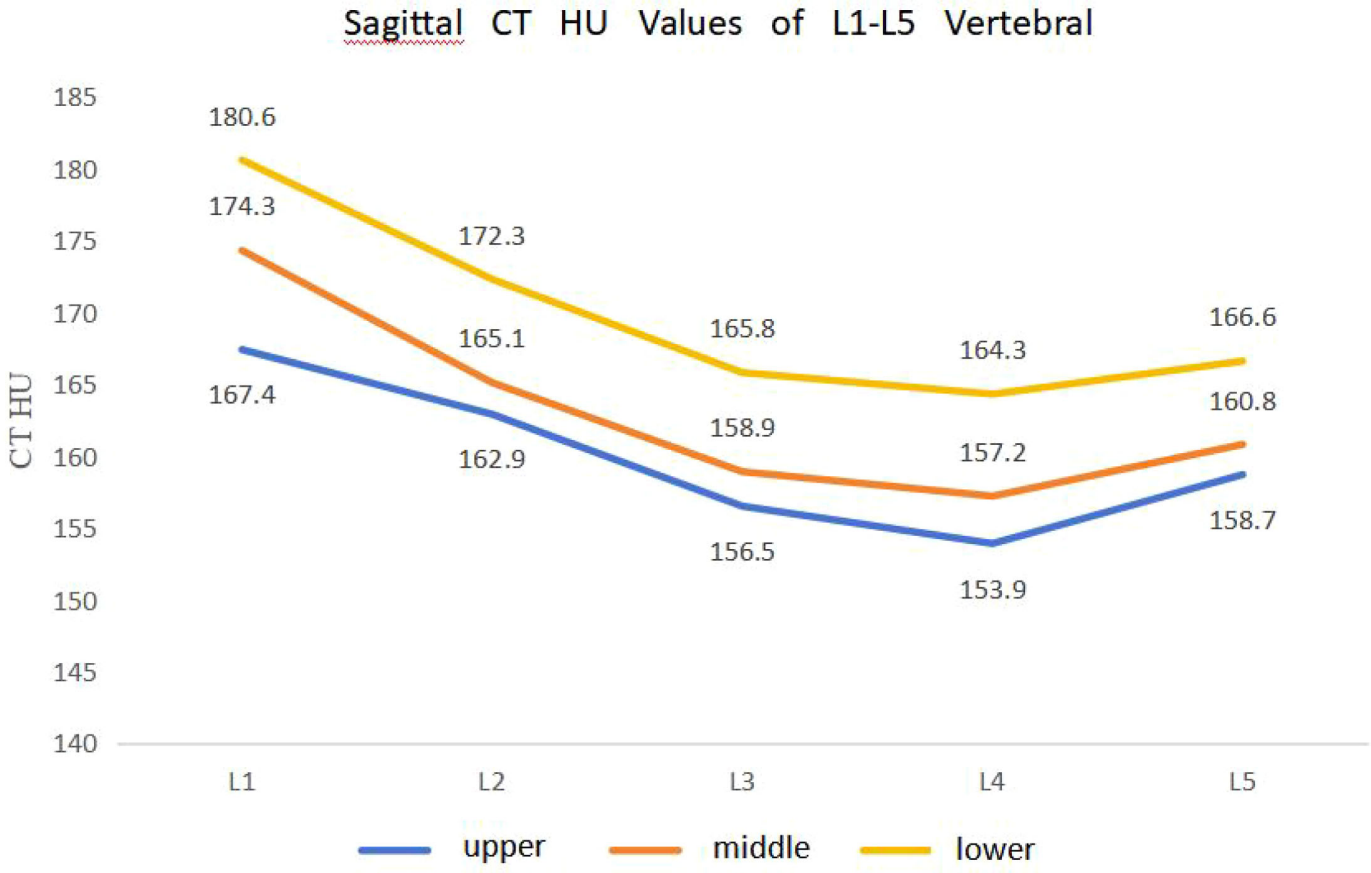
Line graph of sagittal CT HU values for L1-L5 vertebral bodies.

CT values for the upper, middle, and lower thirds of each vertebral body in the sagittal plane consistently showed an increasing trend: upper 1/3 < middle 1/3 < lower 1/3 (**Table 3**; **Figure 4**). Within the same segment, CT values in the lower third were significantly higher than those in the upper third (P < 0.05).

**Table 3:** Sagittal CT HU values of L1-L5 vertebral bodies (mean ± SD).

Level	Upper third	Middle third	Lower third
L1	167.4 ± 26.6	174.3 ± 21.2	180.6 ± 20.1
L2	162.9 ± 30.4	165.1 ± 29.2	172.3 ± 13.3
L3	156.5 ± 24.6	158.9 ± 17.5	165.8 ± 14.5
L4	153.9 ± 21.2	157.2 ± 13.2	164.3 ± 10.9
L5	158.7 ± 24.2	160.8 ± 13.9	166.6 ± 8.4

#### 3.3.2 Comparison of horizontal quadrants

**Tables 4**–**6** present the CT values for the four quadrants at the upper, middle, and lower third levels. Across all levels and segments, CT values in the anterior quadrants of the vertebral body (especially the anterosuperior region) were lower than those in the posterior quadrants. Taking the upper third of L1 as an example, CT values in the posterior quadrants (3 and 4) (174.8 ± 23.3, 175.3 ± 23.3) were significantly higher than those in quadrants 1 and 2 (anterior) (158.1 ± 26.2, 157.5 ± 30.9) (P < 0.01). The L5 vertebra showed a contrary trend: CT values in quadrants 1 and 2 of the upper third were slightly higher than those in quadrants 3 and 4, but the difference did not reach statistical significance.

**Table 4:** CT HU values for upper third horizontal quadrants of L1–L5 (mean ± SD).

Level	Upper Q1 (LA)	Upper Q2 (RA)	Upper Q3 (LP)	Upper Q4 (RP)
L1	158.1 ± 26.2	157.5 ± 30.9	174.8 ± 23.3	175.3 ± 23.3
L2	152.3 ± 28.7	151.7 ± 26.7	167.5 ± 18.0	169.5 ± 18.9
L3	151.5 ± 26.8	149.3 ± 23.9	163.1 ± 37.6	166.9 ± 30.4
L4	146.7 ± 17.8	148.8 ± 13.5	161.7 ± 14.5	162.2 ± 8.5
L5	152.0 ± 27.7	156.9 ± 21.8	164.0 ± 26.9	167.2 ± 24.5

**Table 5:** CT HU values for middle third horizontal quadrants of L1–L5 (mean ± SD).

Level	Middle Q1 (LA)	Middle Q2 (RA)	Middle Q3 (LP)	Middle Q4 (RP)
L1	165.1 ± 19.7	164.8 ± 17.0	180.3 ± 14.0	181.7 ± 13.2
L2	159.2 ± 23.0	158.4 ± 22.6	172.3 ± 16.3	174.8 ± 12.8
L3	151.3 ± 25.9	153.5 ± 29.1	165.1 ± 29.1	167.6 ± 29.4
L4	150.8 ± 13.3	152.8 ± 17.4	163.8 ± 22.0	161.0 ± 18.0
L5	157.1 ± 21.4	154.1 ± 15.7	167.1 ± 23.7	168.8 ± 18.1

**Table 6:** CT HU values for lower third horizontal quadrants of L1–L5 (mean ± SD).

Level	Lower Q1 (LA)	Lower Q2 (RA)	Lower Q3 (LP)	Lower Q4 (RP)
L1	173.9 ± 22.5	172.8 ± 22.4	183.2 ± 16.2	185.5 ± 16.1
L2	170.5 ± 16.3	167.5 ± 20.5	178.3 ± 16.0	179.2 ± 26.4
L3	160.5 ± 24.3	159.2 ± 26.7	172.9 ± 21.8	169.1 ± 17.3
L4	160.2 ± 22.1	158.2 ± 26.1	168.2 ± 18.6	168.6 ± 20.1
L5	161.8 ± 25.8	162.3 ± 30.0	174.0 ± 20.4	172.7 ± 20.0

Q, Quadrant; LA, Left Anterior; RA, Right Anterior; LP, Left Posterior; RP, Right Posterior.

#### 3.3.3 Longitudinal comparison between vertebral levels

Regardless of sagittal or horizontal partitioning, CT values across different vertebral levels generally followed a similar pattern: L1 highest, followed by L2, then L5, with L3 and L4 being the lowest (**Tables 3**–**6**). For example, at the upper third level, the overall mean CT value for L1 was 167.4 HU, L2 was 162.9 HU, L3 was 156.5 HU, L4 was 153.9 HU, and L5 was 158.7 HU.

## 4 Discussion

### 4.1 Morphological evolution of lumbar vertebral bodies and its biomechanical significance

This study found a significant progressive increase in both superior and inferior endplate widths from L1 to L5. Specifically, the superior endplate width increased from 41.8 ± 1.0 mm at L1 to 50.7 ± 2.4 mm at L5 (a 21.3% increase), while the inferior endplate width increased from 44.8 ± 1.1 mm at L1 to 52.4 ± 0.6 mm at L5 (a 17.0% increase). This is consistent with the findings of Liu et al. (2018) and Han et al. (2023), as well as the anatomical study by Shrestha (2022), which reported a mean L5 width of 42.59 mm in a Nepalese population, and the MRI-based morphometric study by Nişancı and Yıldız (2026) at the L4-L5 level. This morphological change directly reflects the gradient increase in mechanical load. L1 primarily bears the weight above the thorax, while L5, as the “keystone” of the lumbosacral junction, endures the highest cumulative load. Increasing the endplate area by approximately 20% from L1 to L5 effectively disperses compressive stress and enhances stability (Hussein et al., 2013; Ashish et al., 2023).

The observed patterns in vertebral body height collectively form the morphological basis of physiological lumbar lordosis. Our data revealed that at L1 and L2, the posterior height exceeded the anterior height (e.g., L1: 27.2 ± 1.3 mm vs. 25.1 ± 1.4 mm, a difference of 2.1 mm). At L3, the anterior and posterior heights were approximately equal (27.1 ± 1.4 mm vs. 27.3 ± 2.2 mm). At L4 and L5, the anterior height exceeded the posterior height (L5: 27.7 ± 0.6 mm vs. 24.6 ± 2.5 mm, a difference of 3.1 mm). This wedge - shaped variation, from being posteriorly higher at upper lumbar levels to anteriorly higher at lower levels, is consistent with the MRI study by Hegazy et al (Hegazy and Hegazy, 2014) and the anatomical measurements by Hou et al. (2013). It enables the spine to form an elastic “S”-shaped curve in the sagittal plane, enhancing shock absorption capacity and maintaining balance (Tan et al., 2004; Kaur et al., 2016).Notably, the middle height of each vertebra was consistently less than both the anterior and posterior heights (e.g., at L1: VBHm 23.5 ± 1.3 mm vs. VBHa 25.1 ± 1.4 mm and VBHp 27.2 ± 1.3 mm), resulting in a slightly concave central region that may optimize trabecular bone load transmission (Kunkel et al., 2011; Liu et al., 2015).

Regarding endplate cortical thickness, our study found that the mean thickness of the anterior 1/3 of the superior endplate (0.8 ± 0.1 mm to 0.8 ± 0.2 mm) was significantly less than that of the posterior 1/3 (0.9 ± 0.2 mm to 1.0 ± 0.2 mm) across all lumbar levels (P < 0.05). This 10 - 20% thickness difference, which is consistent across all lumbar levels, suggests that the anterior region is biomechanically weaker and more susceptible to fracture. (Zhao et al. 2009) similarly reported that the anterior endplate was often the first to fail under compression. Lumbar physiological lordosis results in greater compressive stress on the anterior vertebral edge during daily weight - bearing, with the anterior column bearing approximately 80% of the stress (Li et al., 2024). Epidemiological data further support this vulnerability: postmenopausal women with greater vertebral body height heterogeneity have been shown to be at significantly higher risk for incident lumbar fractures, independent of areal BMD measured by DXA (Roux et al., 2016).

### 4.2 Heterogeneity of intravertebral BMD distribution and its clinical significance

This study demonstrated that CT values within each vertebral segment consistently followed an increasing trend from the upper to the lower third. Taking L1 as an example, the mean CT value increased from 167.4 ± 26.6 HU in the upper third to 174.3 ± 21.2 HU in the middle third and 180.6 ± 20.1 HU in the lower third—a gradient of approximately 13 HU (7.9%) from top to bottom. For L4, the gradient was even more pronounced: 153.9 ± 21.2 HU (upper), 157.2 ± 13.2 HU (middle), and 164.3 ± 10.9 HU (lower), representing a 10.4 HU (6.8%) increase. This craniocaudal density gradient, observed across all five lumbar levels, is consistent with the findings of Wang et al. (2022) and Li et al. (2011), and confirms the inhomogeneity of intravertebral BMD distribution, as also reported by Guan et al. (2023) in patients with osteoporotic vertebral compression fractures. This regional heterogeneity has also been characterized using high - resolution peripheral quantitative CT (HR - pQCT), which revealed that the superior vertebral body exhibits not only lower density but also fewer and more widely separated trabeculae compared to the inferior region (Liu et al., 2018).

Longitudinal comparison across vertebral levels revealed that CT values roughly followed the pattern L1 > L2 > L5 > L3 > L4. Specifically, at the upper third level, the overall mean CT value was highest at L1 (167.4 ± 26.6 HU), followed by L2 (162.9 ± 30.4 HU), then L5 (158.7 ± 24.2 HU), with L3 (156.5 ± 24.6 HU) and L4 (153.9 ± 21.2 HU) being the lowest. The difference between L1 and L4 (13.5 HU, 8.1%) was statistically significant (P < 0.05). The highest value at L1 may relate to relatively stable stress at the thoracolumbar junction. L3 and L4, as the center of lumbar motion, endure the greatest dynamic loads, potentially leading to relatively lower BMD due to accelerated bone turnover. Although L5 bears substantial compressive force, its larger size and frequent reactive osteosclerosis may explain its higher CT values compared to L3 and L4. Liu et al. (2005) measured 168 healthy males and found a gradual decrease in BMD from L1 to L4 across all age groups, which is consistent with our segmental comparison.

Most importantly, this study identified that the anterosuperior region (anterior quadrant of the upper third) exhibited the lowest CT values across all levels. Taking L1 as an example, CT values in the posterior quadrants of the upper third (Q3: 174.8 ± 23.3 HU, Q4: 175.3 ± 23.3 HU) were significantly higher than those in the anterior quadrants (Q1: 158.1 ± 26.2 HU, Q2: 157.5 ± 30.9 HU) (P < 0.01).This anterior-posterior difference of approximately 16–17 HU (10%) was consistently observed across all levels from L1 to L4. For L5, however, the pattern was less pronounced, with anterior quadrant values (Q1: 152.0 ± 27.7 HU, Q2: 156.9 ± 21.8 HU) only slightly lower than posterior ones (Q3: 164.0 ± 26.9 HU, Q4: 167.2 ± 24.5 HU), and the difference did not reach statistical significance.

This distribution pattern closely matches the predilection site for clinical osteoporotic vertebral compression fractures, which commonly occur in the superior and anterior parts of the vertebra. Elarjani et al (Zhang et al., 2023) noted that a mid-sagittal CT value below 100 HU indicates poor bone quality; in our study, the anterosuperior region of L4, the level with the lowest overall density, approached 146.7 ± 17.8 HU, still above this threshold in healthy adults, but suggesting that in osteoporotic patients this region may fall well below 100 HU.

From a biomechanical modeling perspective, incorporating this regional heterogeneity of material properties-rather than assuming uniform bone density-significantly improves the accuracy of finite element predictions of vertebral strength and failure location (Oefner et al., 2022).

### 4.3 Clinical significance and integration with current evidence

This study establishes a detailed database of lumbar vertebral morphology and regional bone mineral density (BMD) distribution in healthy Chinese adults. The quantitative data we present-endplate widths ranging from 41.8 mm (L1 superior) to 52.4 mm (L5 inferior), anterior - posterior height differences ranging from -2.1 mm at L1 (posterior > anterior) to +3.1 mm at L5 (anterior > posterior), and regional CT value variations of up to 17 HU (10%) between anterior and posterior regions—provide essential reference values for spinal surgical planning.

In interbody cage design, the progressive increase in endplate width from L1 to L5 (21.3% for superior endplate, 17.0% for inferior endplate) must be fully considered. A cage sized for L1 (approximately 42 mm in width) would be substantially undersized for L5 (approximately 51 mm), leading to inadequate endplate coverage and an increased risk of subsidence. Due to the thinner anterior endplate cortex (0.8 mm vs. 0.9 - 1.0 mm posteriorly, a 10 - 20% difference), excessive distraction or compression during surgery should be avoided to prevent endplate breach.

The clinical relevance of CT-based bone density assessment is increasingly recognized: recent studies have demonstrated that vertebral Hounsfield unit values correlate well with DXA T-scores and can effectively predict complications such as screw loosening, cage subsidence, and adjacent segment fractures (Zhou et al., 2021; Zhang et al., 2023). Specifically, a mean lumbar CT value below 100 HU has been proposed as a threshold for considering cement augmentation or more robust fixation techniques (Elarjani et al., 2021). Our finding that the anterosuperior region exhibits the lowest values (often approaching or falling below such thresholds even when global vertebral density appears acceptable) underscores the importance of regional assessment.

In osteoporotic patients, the anterosuperior region of the vertebral body-particularly at L3 and L4, where overall CT values were lowest (L4 upper third: 153.9 ± 21.2 HU) and the anterior - posterior gradient was most pronounced-represents a critical area for bone cement augmentation or screw fixation. Additionally, the observed regional heterogeneity has direct implications for minimally invasive procedures such as percutaneous vertebroplasty and kyphoplasty. Optimal cement delivery should target the anterosuperior “stress - weak zone” to restore anterior column support and prevent recurrent collapse, rather than simply filling the central cavity (Zhou et al., 2021).

### 4.4 Limitations and future directions

This study has several limitations: ① It is a single-center, cross-sectional study with a modest sample size (n=100) and uneven age distribution (e.g., only 20 participants in the 41–50 age group). No *a priori* power analysis was conducted, which limits the generalizability of subgroup findings. ② Sex−stratified analysis was not performed, despite a balanced male/female sample. Given known sex differences in both vertebral morphology and BMD (Roux et al., 2016), future studies with larger samples should specifically examine male−female differences in regional density patterns. ③ It did not account for the influence of factors like height, weight, or body mass index on morphology. ④ Measurements were performed manually. Only intraobserver reproducibility was assessed (ICC>0.90); interobserver reliability was not evaluated. Automated deep learning−based segmentation methods could be applied in future studies to further improve reproducibility and efficiency (Baur et al., 2022). ⑤ No direct comparison with DXA was performed. CT values are not equivalent to DXA−derived BMD. While CT value is a convenient surrogate, it is influenced by scanner type, tube voltage, and contrast enhancement. We standardized these parameters in our protocol, but phantomless calibration techniques would enhance cross−site comparability in future multi−center studies (Abdullayev et al., 2018). ⑥ The study did not validate the CT−derived regional heterogeneity against biomechanical testing or fracture outcomes.

Future research should expand the sample size, conduct multi-center studies, and integrate fracture line mapping techniques to verify the correspondence between vulnerable regions and fracture morphology. Prospective longitudinal studies are needed to determine whether the regional BMD heterogeneity observed in healthy adults predicts incident fracture risk independently of global BMD.

## 5 Conclusion

The morphological changes of lumbar vertebral bodies from L1 to L5 follow a clear pattern: endplate width progressively increases to accommodate axial load; the relationship between anterior and posterior vertebral heights transitions from posterior height exceeding anterior height to the opposite, forming the structural basis of physiological lordosis. The anterior cortex of the superior endplate is thinner than the posterior cortex. The distribution of CT values within the vertebral body exhibits significant heterogeneity, consistently showing upper 1/3 < middle 1/3 < lower 1/3 in each vertebra, with anterior regions lower than posterior regions. Values are relatively higher at L1-L2 and L5, and lower at L3-L4. This distribution pattern renders the anterosuperior region a stress-weak zone, highly consistent with the predilection site for osteoporotic fractures. The morphological and densitometric database established in this study provides a refined basis for individualized treatment in spinal surgery and biomechanical research.

## Data Availability

The original contributions presented in the study are included in the article/supplementary material. Further inquiries can be directed to the corresponding author.
